# Biochemical Properties and Structure Analysis of a DAG-Like Lipase from *Malassezia globosa*

**DOI:** 10.3390/ijms16034865

**Published:** 2015-03-04

**Authors:** Huan Xu, Dongming Lan, Bo Yang, Yonghua Wang

**Affiliations:** 1 College of Light Industry and Food Sciences, South China University of Technology, Guangzhou 510640, China; E-Mails: xuhuanscut@163.com (H.X.); dmlanscut.edu.cn (D.L.); 2 School of Bioscience and Bioengineering, South China University of Technology, Guangzhou 510006, China; E-Mail: yangbo@scut.edu.cn

**Keywords:** DAG-like lipases, *Malassezia globosa*, cold active enzyme, glycosylation, structure analysis

## Abstract

Diacylglycerol (DAG)-like lipases are found to play an important role in the life sciences and industrial fields. A putative DAG-like lipase (*Mg*MDL2) from *Malassezia globosa* was cloned and expressed in recombinant *Pichia pastoris*. The recombinant *Mg*MDL2 was expressed as a glycosylated protein and purified into homogeneity by anion exchange chromatography. The activity of recombinant *Mg*MDL2 was optimal at 15 °C and pH 6.0, and it keeps over 50% of relative activity at 5 °C, suggesting that *Mg*MDL2 was a cold active lipase. *Mg*MDL2 retained over 80% of initial activity after incubation at 30 and 40 °C for 2.5 h, but it was not stable at 50 °C. Incubation of methanol and ethanol at a concentration of 30% for 2 h did not affect the recombinant enzyme activity, while metal ions, including Ca^2+^, Mn^2+^ and Ni^2+^, sharply inhibited the *Mg*MDL2 activity at 5 mM by 42%, 35% and 36%, respectively. *Mg*MDL2 exhibited a preference for medium chain-length esters with highest activity toward *p*-nitrophenyl caprylate, while it was active on mono- and diacylglycerol but not on triacylglycerol, indicating that it was a typical DAG-like lipase. By homology modeling, Phe278 was predicted to be involved in the preference of *Mg*MDL2 for monoacyl- and diacyl-glyceride substrates, but not triglycerides.

## 1. Introduction

DAG-like lipases, distinct from common triacylglycerol (TAG) lipases, are enzymes that exhibit specific activity on mono- and diacylglycerol (MAG and DAG) but not on TAG. They play an important role in regulating the physiological function in mammals. For example, they are found to be involved in the biosynthesis of the endocannabinoid 2-arachidonoylglycerol, an endogenous agonist of the CB1 receptor, which mediate neuronal communication [1]. DAG-like lipases are considered as a valuable target to develop therapeutic inhibitor for pain, inflammation, degenerative diseases, tissue injury, and cancer [2]. Besides that, they also are potential biocatalysts that could be used for modification of oil and fats [3,4].

To date, few lipases including *Penicillium camembertii* (*P. camembertii*) U-150 [5], *Penicillium cyclopium* (*P. cyclopium*) [6], *Fusarium* sp. YM-30 [7], *Aspergillus oryzae* (*A. oryzae*) [8,9] and *Malassezia globosa* (*M. globosa*) [10], have been experimentally characterized as DAG-like lipases. Although sequence comparison analysis shows relatively low sequence identities to the TAG lipases, DAG-like lipases from *P. camembertii* U-150, *A. oryzae* and *M. globosa* are found to belong to *Rhizomucor mihei* (*R. mihei*) lipase like family (LED ID: abH23.01) at lipase engineering database by sequence analysis [11]. Both crystal structures from *M. globosa* (named as SMG1) and *P. camembertii* have been solved [12,13]. However, limited information was obtained from the *P. camembertii* lipase structure because the quality of the crystal data was affected by the twinning of the crystals. It is found that they have a canonical α/β hydrolase fold core which is a characteristic of all TAG-lipases family members [12]. By structural modeling analysis, a unique “bridge-like” structure was found to exist on the top of catalytic site of DAG-like lipases, which maybe limit the size of substrate allowed to enter the active site. This may contribute to their unique substrate selectivity [11].

TAG-lipases have been intensive investigation and many of them were commercial available, while the DAG-like lipases were rarely studied due to the limited number of them reported. To explore the industrial application of DAG-like lipases and fully understanding their molecular basis of substrate specificity, novel DAG-like lipases with unique properties need to be explored. In our previously study, a DAG-like lipase (SMG1) from *M. globosa* has been characterized by our lab [3]. In this study, a putative DAG-like lipase (XP_001732206, Name as *Mg*MDL2), which showed high sequence identity (67%) with that of SMG1, has been cloned, and its biochemical properties such as substrate selectivity, glycolysation, effect of pH, temperature, organic solvents and metal ions on enzyme activity, were explored. Moreover, the molecular basis for the substrate specificity of *Mg*MDL2 was discussed.

## 2. Results and Discussion

### 2.1. Sequence Analysis of MgMDL2

**Figure 1:**
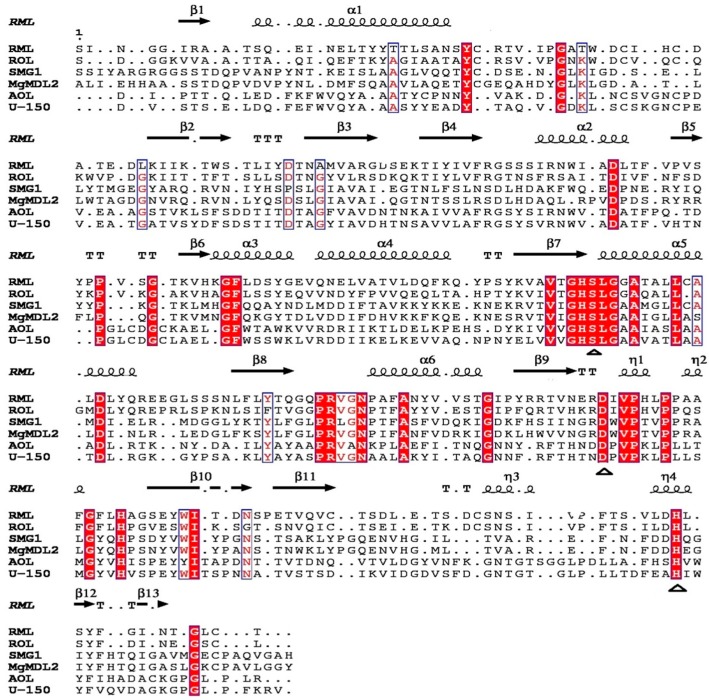
Multiple sequence alignment of *Mg*MDL2. The lipases sequence included U-150 from *P. camembertii* U-150 (PDB: 1TIA_A), AOL from *A. oryzae* (XP_001823459 in GenBank), PCL from *P. cyclopium* (ADE87963 in GenBank), SMG1 from *M. globosa* (PDB: 3UUE), *Mg*MDL2 from *M. globosa* (XP_001732206 in GenBank), RML from *R. miehei* (PDB ID: 4TGL) and ROL from *R. oryzae* (PDB ID: 1TIC). Identical residues were marked with red background and the highly conserved residues were showed as red font. The catalytic triads are indicated with triangle.

The *MgMDL2* gene is 915 bp in length, encoding a 304 aa protein. A deduced signal peptide region of 19 amino acids was found by analysis of SignalP 4.1. *Mg*MDL2 has three Asn-Xaa-Thr/ser motifs (Asn102, 161 and 253) which are considered as potential glycosylation sites. According to the multiple alignment analysis, *Mg*MDL2 was found to contain the characteristic active site of the α/β hydrolase fold enzyme. Catalytic residues (serine171, aspartate228, and histidine281) form the putative catalytic triad, and the nucleophilic serine residue locates at a highly conserved G-H-S-L-G pentapeptide motif (Figure 1). *Mg*MDL2 exhibited high sequence identity with the SMG1 (67%). It shares only 20%, 20% and 19% sequence identity with those of DAG-like lipases from *P. camembertii* U-150 [5], *P. cyclopium* [6] and *A. oryzae* [9] (Figure 1), respectively, while *Mg*MDL2 has a relative high sequence identity with that of TAG-lipases from *R. miehei* (28%) [14] and *Rhizopus oryzae* (*R. oryzae*) (24%) [15].

### 2.2. Expression and Purification of MgMDL2

**Figure 2:**
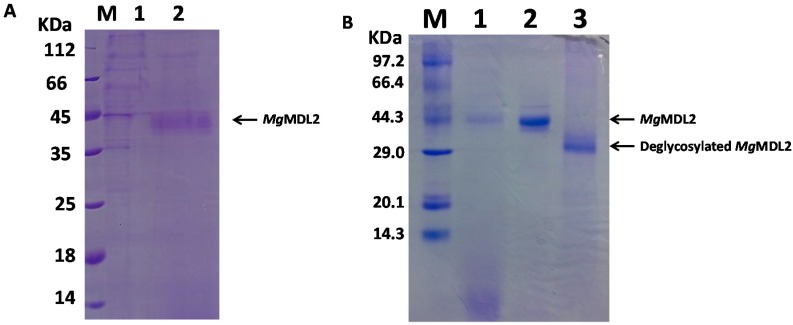
SDS-PAGE analysis of purification and deglycosylation of *Mg*MDL2. (**A**) Detection of expression of *Mg*MDL2. Lane M: marker; Lane 1: Supernatants of control *P. pastoris* X-33; Lane 2: Supernatants of recombinant *P. pastoris* X-33 having pGAPZαA-*mgmdl2*; (**B**) Purification and deglycosylation of *Mg*MDL2. Lane 1: Supernatant of fermentation at 72 h; Lane 2: purified *Mg*MDL2; Lane 3: Deglycosylated *Mg*MDL2.

The recombinant enzyme was purified by anion exchange chromatography into homogeneity with specific activity of 43.61 U/mg (*pnp*-caprylate as substrate). SDS-PAGE analysis of the purified *Mg*MDL2 shows that it had an apparent molecular mass of 44 kDa (Figure 2, lane 2), which was larger than the deduced one (31 kDa). *N*-glycosylation is a major kind of protein posttranslational modification in *Pichia pastoris* (*P. pastoris*), and 70%–90% of the Asn residues in potential *N*-glycosylation sites (Asn–Xaa–Ser/Thr) will be modified by glycosylation [16]. *Mg*MDL2 has three potential *N*-glycosylation sites; therefore it may be modified by *N*-linked glycosylation in yeast. To confirm that, glycopeptidase F which can specifically hydrolyze *N*-glycans from glycoprotein was used for treating the purified *Mg*MDL2. After deglycosylation reaction, a protein band of 31 kDa was observed (Figure 2, lane 3).

### 2.3. Biochemical Properties of MgMDL2

To investigate whether *Mg*MDL2 was a true DAG-like lipase and the potential as a biocatalyst in industry application, the biochemical properties of purified *Mg*MDL2 were studied.

#### 2.3.1. Effect of Temperature on Lipase Activity and Thermostability

**Figure 3:**
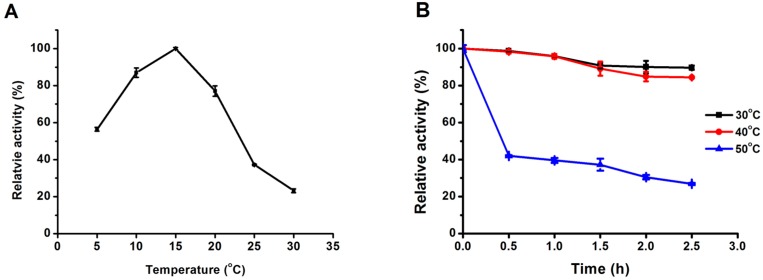
Effect of temperature on activity (**A**); and stability (**B**) of *Mg*MDL2. (**A**) The purified *Mg*MDL2 was assayed at different temperatures (5–30 °C). Activities are displayed as percentages of the maximum activity. Values are means ± S.D. from three independent experiments; (**B**) The enzyme was assayed after incubation in various temperatures (30, 40 and 50 °C) for 2.5 h. Activity is displayed as percentages of the initial activity.

The optimum temperature of *Mg*MDL2 was determined using *p*np caprylate as substrate. Recombinant *Mg*MDL2 showed highest activity at 15 °C (Figure 3A). Even at 5 °C, *Mg*MDL2 kept over 50% activity.

The thermostability of *Mg*MDL2 was investigated at three different temperatures (30, 40 and 50 °C) with increasing incubation time up to 2.5 h. As shown in Figure 3B, the *Mg*MDL2 was still active after incubation at 30 and 40 °C, which retained above 89.6% and 84.4% of the initial activity, respectively. However, only 26% of initial activity was retained following incubation for 2.5 h at 50 °C.

#### 2.3.2. Effect of pH on Lipase Activity and Stability

**Figure 4:**
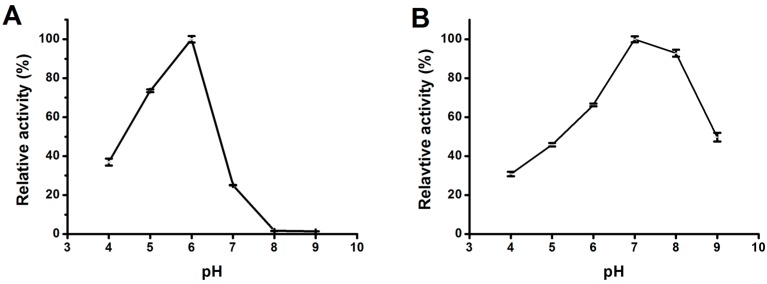
Effect of pH on the activity (**A**); and stability (**B**) of *Mg*MDL2. Activities are shown as percentages of the maximum activity. Values are means ± S.D. from three independent experiments.

The activity of *Mg*MDL2 was measured over a pH range of 4.0–9.0. As shown at Figure 4A, *Mg*MDL2 showed activity within a wide pH range (pH 4.0–7.0) with an optimal activity at pH 6.0 while it was totally inactive at pH 8.0 and 9.0. *Mg*MDL2 found to be stable over pH 6.0–8.0 with residual activity over 60% of initial activity (Figure 4B).

#### 2.3.3. Effect of Chemicals and Organic Solvents on *Mg*MDL2 Activity

The influence of organic solvents and metal ions on the activity of *Mg*MDL2 was investigated and the results are summarized in Table 1. The enzyme was inhibited by isopropanol by 59% of initial activity, whereas it was not affected by methanol and ethanol, and weakly inhibited by acetone (retaining 85% of initial activity). To determine the effect of metal ions on *Mg*MDL2 activity, the enzyme activities were tested in the presence of 5 mM of various metal ions. All metal ions tested can inhibit enzyme activity. Among them, Ca^2+^, Mn^2+^ and Ni^2+^ sharply decreased the activity to 42%, 35% and 36% of initial activity, respectively.

**Table 1:** Effects of various reagents on *Mg*MDL2 activity.

Reagents	Relative Activity (%) ^a^	
	5 mM	30% (*v*/*v*)
Control	100	100
Ni^2+^	36 ± 1	-
Zn^2+^	61 ± 4	-
Mn^2+^	35 ± 4	-
Mg^2+^	53 ± 4	-
Fe^2+^	48 ± 2	-
Ca^2+^	42 ± 1	-
EDTA	68 ± 1	-
Methanol	-	98 ± 2
Ethanol	-	97 ± 4
Acetone	-	85 ± 4
Isopropanol	-	41 ± 2

- Not determined; ^a^ Data are presented mean ± standard deviation.

#### 2.3.4. Substrate Specificity of *Mg*MDL2

**Figure 5:**
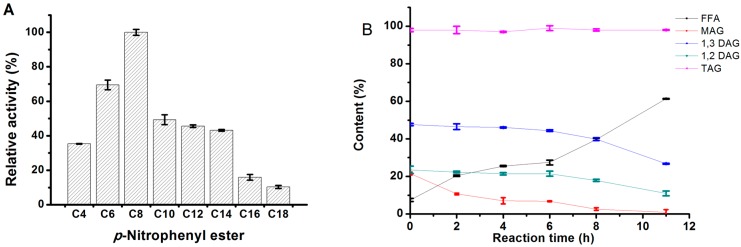
Determination of the substrate specificity of *Mg*MDL2. (**A**) Activities on each *p*np ester are expressed as the percentage of *p*np caprylate. Values are means ± S.D. from three independent experiments; (**B**) Time course of hydrolysis of acylglycerols by *Mg*MDL2. The hydrolysis reaction were performed using DAG-rich oil (23.47% of 1,2-DAG, 47.60% of 1,3-DAG, 21.51% of MAG and 7.42% of FFA) or TAG (camellia oil, 98%) as substrate. The reaction products contents were analyzed by HPLC and showed in same curve.

The substrate preference of the *Mg*MDL2 was firstly investigated using *p*-nitrophenyl (*p*np) ester with acyl chains of various lengths. As shown in Figure 5A, *Mg*MDL2 has a strong preference for the hydrolysis of the ester bonds of medium chain-length esters with highest activity toward *p*np caprylate (C8), while *Mg*MDL2 displayed low activity toward the long chain-length esters (C16 and C18 acyl group).

For acylglycerol substrate, *Mg*MDL2 was active toward the MAG and DAG, but inactive toward TAG (Figure 5B), indicating that the *Mg*MDL2 was a true DAG-like lipase.

### 2.4. Molecular Basis for Substrate Selectivity of MgMDL2

**Figure 6:**
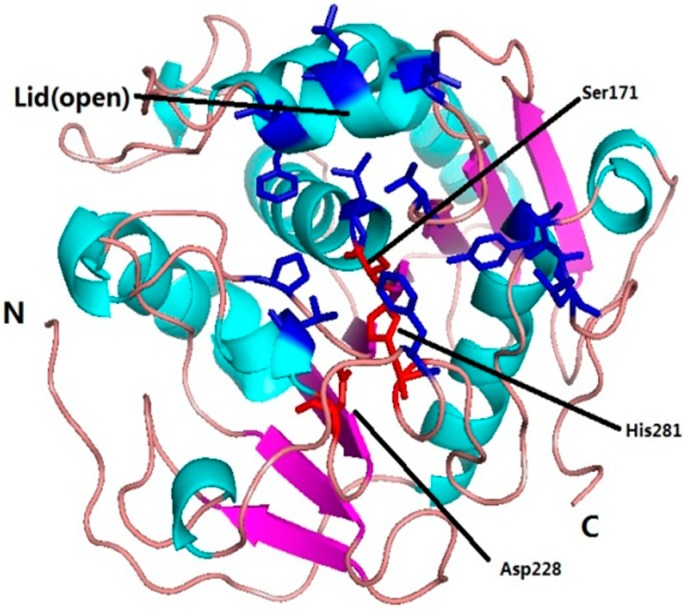
Cartoon view of the modeled *Mg*MDL2 in the open conformation. The catalytic pocket and the residues constituted the oxyanion hole highlighted in blue, while the catalytic triads were colored red.

The modeling structure in open conformation led to the catalytic triad Ser171-Asp228-His281 exposing to the solvent (Figure 6). In the open conformation, the oxyanion hole was formed by residue Thr101 and Leu172 .The residues Trp53, Leu106, Leu110, Ala113, Phe138, Leu172, Pro201, Val230, Phe278, Ser293 and Leu294 were found to constitute the catalytic pocket.

**Figure 7:**
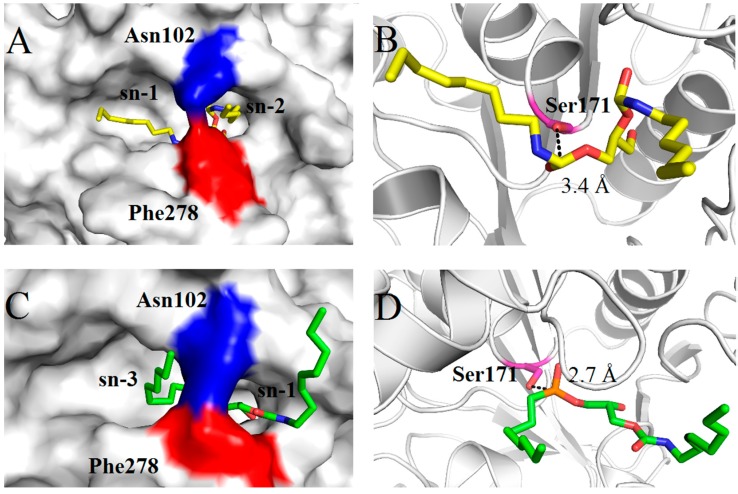
The 3-D structures of *Mg*MDL2-DAG analogue complexes. (**A**) Surface view of the optimized *Mg*MDL2-1, 2-DAG analogue; (**B**) A close-up view showing the distance from 1, 2-DAG analogue carboxyl carbon to Oγ of Ser171 in *Mg*MDL2; (**C**) Surface view of the optimized *Mg*MDL2-1, 3-DAG analogue; (**D**) A close-up view showing the distance from 1, 3-DAG analogue phosphorus atom (substitution of carboxyl carbon) to Oγ of Ser171 in *Mg*MDL2.

In the optimized *Mg*MDL2-1, 2-DAG analogue complex structure (Figure 7A), the distance between carboxyl carbon and Oγ of Ser171 is 3.4 Å (Figure 7B). While in the best conformation of *Mg*MDL2-1, 3-DAG analogue complex structure(Figure 7C) ,the distance between phosphorus atom (substitution of carboxyl carbon) and Oγ of Ser171 is 2.7 Å (Figure 7D). As shown in Figure 7A,C, the catalytic pocket was divided by the side chain of Asn102 and Phe278 into two separated parts, exposed a groove and a narrow tunnel. In *Mg*MDL2-1, 2-DAG analogue complex structure, the sn-1 alkyl chain lies in the exposed groove while the sn-2 moiety of substrate inserts into the narrow tunnel. However in *Mg*MDL2-1, 3-DAG analogue complex structure , the sn-1 moiety of substrate inserts in the narrow tunnel and the sn-3 alkyl chain lies in the exposed groove. Both in *Mg*MDL2-1, 2- and 1,3-DAG analogue complex structures the Phe278 projects its side chain toward active site, thus the catalytic pocket does not have enough space to accommodate the third chain of TAG, and therefore *Mg*MDL2 can only catalyze the MAG and DAG but not TAG. This observation was similar with that of DAG-like lipase from *P. cyclopium* and *A. oryzae* [11,17].

In this study, we described cloning, expression and biochemical characterization of a putative DAG-like lipase from *M. globosa*. Recombinant *Mg*MDL2 was found to be a glycosylated protein. It is reported that recombinant proteins having *N*-glycosylation sites (Asn–Xaa–Ser/Thr) produced in *P. pastoris* are often modified by glycosylation [18]. Despite increase of the molecular mass of recombinant proteins, glycosylation is an important post-translational modification that can affect proteins folding, stability, activity and half-life [19].

*Mg*MDL2 showed highest activity at 15 °C, and it retained more than 50% activity at 5 °C. These properties were similar with that of cold active lipolitic enzyme reported in literatures [20,21]. Although cold active enzymes have many potential applications in industry, most of them have been reported to be unstable at high temperature. Lipase from *Psychrobacter* sp. strain in antarctic sea water completely lost its activity by incubation at 40 and 50 °C for 20 min [22]. h1Lip1 from uncultured bacteria of marine sediment was unstable at 25 °C and its half-life at 40 °C was less than 5 min [23]. M37 lipase from *Photobacterium lipolyticum* sp. was inactive after heat treatment (above 35 °C) for 20 min [24]. In contrast, *Mg*MDL2 was stable after incubation of 30 and 40 °C for 2.5 h, retaining more than 80% original activity.

*Mg*MDL2 was found to be stable in the presence of methanol and ethanol at a concentration of 30% for 2 h, indicating that *Mg*MDL2 is a solvent tolerant enzyme. *Mg*MDL2 was sensitive toward metal ions, such as Ca^2+^, Mn^2+^ and Ni^2+^, which hardly decrease its activity. In contrast, Ca^2+^ and Mn^2+^ was reported to simulate the activity of lipase from *Bacillus sphaericus* 205y [25], *Pseudomonas aeruginosa* LX1 [26] and *Fusarium solani* N4-2 [27]. The lipase lost 32% of its activity in presence of 5 mM EDTA, indicating that *Mg*MDL2 might be a metallohydrolase. Structural analysis of *Mg*MDL2 showed that Phe278 might involve in unique substrate selectivity of *Mg*MDL2. The position and orientation of the phenylalanine seem to be a common phenomenon to DAG-like lipases. For DAG-like lipase from *P. cyclopium*, Phe256 was predicted to play an important role in substrate selectivity [17], while “bridge-like” structure constituted by two bulky residues (W89 and F257) on the top of catalytic site in *A. oryzae* lipase, which prevented substrates with large size from entering the active site [11].

## 3. Experimental Section

### 3.1. Strains, Plasmids, Chemicals and Materials

*Escherichia coli* Top10 (Invitrogen, Carlsbad, CA, USA) was used as cloning host. The plasmid pGAPZαA and *P*. *pastoris* X-33 strain (Invitrogen, Carlsbad, CA, USA) are used for gene cloning and expression, respectively. The *p*-nitrophenol and *p*-nitrophenol (*p*np) ester derivates were purchased from Sigma-Aldrich. *n*-hexane and 2-propanol were of HPLC grade from Kermel Chemical Reagent Co., Ltd. (Tianjin, China).

DAG-rich acylglycerols used in this study were synthesized by enzymatic esterification of glycerol and FFAs in our laboratory. At first, free fatty acids (FFAs) were produced by enzymatic hydrolysis of camellia oil using Palatase 20000L (Novo Nordisk A/S, Bagsvaerd, Denmark), and then the FFA was further purified by molecular distillation. *P. camembertii* lipase, a DAG-like liapse, was used for synthesis of DAG and MAG by esterification of glycerol and FFAs, and then the products were further purified by molecular distillation. The content of DAG-rich oil (23.47% of 1,2-DAG, 47.60% of 1,3-DAG, 21.51% of MAG and 7.42% of FFA) was analyzed by HPLC. Camellia oil (TAG, 99%) was purchased from the local market in China. Other chemicals were of analytical grade.

### 3.2. Vector Construction and Transformation of P. pastoris

The *MgMDL*2 gene (915 bp) derived from *M. globosa* (GenBank accession number: XM_001732154) was artificially synthesized according to the code usage of *P. pastoris* by Sangon Biotech, Inc. (Shanghai, China). The gene encoding the mature peptide (58 to 915 bp, without signal peptide region) was cloned into pGAPZαA (Invitrogen, Carlsbad, CA, USA) vector pGAPZαA-*Mgmdl*2 was confirmed by DNA sequencing and then linearized by restriction enzyme *Bln* I. The purified linearized DNA was transformed into *P. pastoris* X-33 strain by electroporation. The transformants were selected on YPD (1% (*w*/*v*) yeast extract, 2% (*w*/*v*) peptone and 2% (*w*/*v*) glucose) plates containing Zeocin™ (100 µg/mL) at 30 °C for three days until the colony formed.

### 3.3. Expression and Purification of Recombinant Enzyme

The *P*. *pastoris* X-33 transformants containing the recombinant vector were grown and expressed in YPD medium at 30 °C with shaking of 200 rpm for 72 h. The supernatant of fermentation broth was collected by centrifugation (10,000× *g*, 20 min, 4 °C) and filtered through a 0.45 µm filter membrane. The resulting supernatant of fermentation broth was concentrated and buffer-exchanged to buffer A (20 mM Tris–HCl, pH 8.0, at 4 °C) using a crossflow cassette with a 10 kDa cutoff membrane (Vivaflow 200, Sartorius, Germany). The recombinant *Mg*MDL2 were purified form the fermentation broth by anion exchange chromatography. Sample in Buffer A was loaded into the Q-Sepharose Fast Flow column and washed with a linear ion gradient (300 mL of 0–200 mM NaCl in buffer A). The fraction containing purified lipases were collected and analyzed by 12% SDS-PAGE. Protein concentrations were determined by the BCA Protein Assay Kit (Sangon Biotech, Shanghai Co., Ltd., Shanghai, China).

### 3.4. Biochemical Characterization of Recombinant Enzyme

#### 3.4.1. Enzyme Activity Determination

The enzyme activity was determined by colorimetric method using *p*-nitrophenyl esters as substrate [28,29]. One unit of enzyme activity is defined as the amount of enzyme required to release 1 μmol of *p*-nitrophenol per minute.

#### 3.4.2. Determining Temperature-Optimum of Activity and Thermostability of Lipase

The optimum temperature of the lipase was evaluated at pH 6.0 using *p*np caprylate as substrate. The temperatures were set as 5, 10, 15, 20, 25 and 30 °C.

The thermostability of *Mg*MDL2 was tested by pre-incubating the *Mg*MDL*2* at different temperatures for 2.5 h. In addition, samples were taken at intervals of 30 min for measurement of residual activity under the above assay conditions (pH 6.0, optimum temperature). The temperatures were set as 30, 40 and 50 °C.

#### 3.4.3. Determining pH-Optimum of Activity and pH Stability of Lipase

Optimum pH for the *Mg*MDL2 was determined at 15 °C using *p*np caprylate as substrate. The buffers used in this study included 100 mM sodium citrate, 100 mM citric acid (pH 4 and 5), 100 mM phosphate buffer (pH 6 and 7), 50 mM Tris–HCl (pH 8.0) and 50 mM Gly-NaOH (pH 9.0).

The pH stability of lipase was determined by pre-incubating enzymes in different pH buffers (from pH 4.0 to 9.0) for 12 h at 4 °C, and then the residual activity was determined at 15 °C at pH 6.0.

#### 3.4.4. Substrates Specificity

*p*np esters and acylglycerol were used to analyze the substrates specificity of *Mg*MDL2. *p*np esters with different chain length (C4–C18) was used as substrates (10 mM, dissolved in ethanol). Hydrolytic reaction was performed at 15 °C and pH 6.0.

For hydrolysis of acylglycerol, DAG-rich oil and camellia oil (TAG) were used. The hydrolytic reactions were performed in a 15 mL conical flask with stirring at 200 rpm. The reaction mixture includes water (25% content with respect to oil), purified enzyme (12 U/g, with respect to oil), and the optimal temperature were used. Aliquots (150 μL) of the reaction mixture were periodically withdrawn and then were centrifuged at 10,000× *g* for 3 min to remove the water in the upper layer. Twenty μL of supernatant were diluted in 1 mL of *n*-hexane/2-propanol/methanoic acid (15:1:0.003 *v*/*v*/*v*) for HPLC analysis [30].

#### 3.4.5. Effect of Metal Ions on the Enzyme Activity

The influence of metal ions on the lipase activity was determined in the presence of various metal ions. Enzyme activity was tested at 15 °C and pH 6.0 using *p*np caprylate as substrate. Metal ions including ZnSO_4_, MgSO_4_, FeCl_2_, CaCl_2_, MnSO_4_ and NiCl_2_ at a final concentration of 5 mM were used in this study. The effect of ethylenediaminetetraacetic acid (EDTA) on enzyme activity was also investigated as described as above.

#### 3.4.6. Effect of Organic Solvents on the Enzyme Activity

The effect of various organic solvents (methanol, ethanol, acetone and isopropanol) on lipase activity was determined. The lipase solution was incubated separately with each organic solvent (final concentration 30%, *v*/*v*) at 4 °C for 2 h, and then the residual activity of enzyme was measured at 15 °C and pH 6.0 using *p*np caprylate as substrate.

#### 3.4.7. Detection of Glycolysation Modification

Deglycolysation of purified *Mg*MDL2 was performed by using glycopeptidase F kit (Takara Company, Dalian, China) according to the instruction of manufacturer. Briefly, recombinant *Mg*MDL2 (25 μg) was denatured by incubation at 100 °C for 3 min in a denature buffer. In addition, the resulting *Mg*MDL*2* was mixed with glycopeptidase F (1 mU) in reaction buffer and incubated at 37 °C for 15 h. The reaction product was analyzed by 12% SDS-PAGE.

### 3.5. Sequence and Structure Analysis

#### 3.5.1. Sequence Analysis

Similarity searches were performed with BLAST 2.0 program [31]. Prediction of signal peptide of *Mg*MDL2 was carried out by SignalP 4.1 Server [32].The putative glycosylation sites were searching by NetNGlyc 1.0 Server. Multiple sequence alignments were performed with ClustalW2 [33] and ESPript [34].

#### 3.5.2. Construction of the Structure in the Open Conformation

The core domain of *Mg*MDL2 (residues 20–100, 119–304) was constructed by the crystal structure of SMG1 (PDB ID: 3UUE) [12]. The lid domain in open conformation was homologically modeled based on the crystal structure of *Rhizomucor miehei* lipase (RML) in complex with the diethyl *p*np phosphonate (PDB ID: 4TGL) [14]. All of above processes were performed by the Discovery Studio. VERIFY-3D (Discovery Studio, Accelrys Inc., San Diego, CA, USA) was used to validate the refined model of *Mg*MDL2.

#### 3.5.3. Construction of a Model in Complex with Substrate Analogue

A 1,2- and 1,3-diacylglycerol analogues were extracted from the crystal structure of *Pseudomonas aeruginosa* lipase (PDB: 1EX9) by removing the sn-3 or sn-2 respectively [35]. Molecular dynamics (MD) were carried out to fully relax the steric clashes occurred in this enzyme-substrate analogue complex.

#### 3.5.4. MD Simulations

The MD simulations were performed by the Discovery Studio package. The *Mg*MDL2-1,2- and 1,3-DAG analog complexes were solved in an orthorhombic box with 7940 and 7979 water molecules respectively and simulated with periodic boundary conditions. After twice energy minimizations, the final RMS gradient converged to 0.0001 kcal/(mol × Å). The Particle Mesh Ewald algorithm was applied to the following simulations. The system was heated from 50 to 300 K for 2000 steps (0.001 ps per step) and equilibrated at 300 K for 1000 steps (0.001 ps per step). Production simulations were worked at 300 K for 5000 steps (0.001 ps per step). All the processes used the CHARMM force field. The optimized 3-D structure of *Mg*MDL2-substrate analogues were visualized using the PyMOL software.

## 4. Conclusions

DAG-like lipases are kinds of enzymes that are found to be important in life science and have potential in industrial applications. However, few works have been done on them. Here, the other enzyme (*Mg*MDL2) from *M. globosa* was researched. *Mg*MDL2 was identified to be a typical DAG-like lipase which shows activity on MAG and DAG not on TAG. *Mg*MDL2 was a cold active lipase with optimal activity at 15 °C and it kept over 50% of relative activity at 5 °C. By modeling *Mg*MDL2 in open conformation, a DAG analogue was docked into the catalytic pocket to get the optimized *Mg*MDL2-DAG analogue complex structure. Phe278 was predicted to be involved in substrate specificity of *Mg*MDL2. Here, we have described a novel cold-active DAG-like lipase which is a potential biocatalyst for industrial application such as, oil modification, biodiesel, food, detergent and pharmaceutical industry. Our work also shed some light on understanding the structural-functional relationship of DAG-like lipases.
